# The State Examining and Licensing Board Again

**Published:** 1891-05

**Authors:** 


### THE STATE EXAMINING AND LICENSING BOARD AGAIN.

SOME one has prayed to be delivered from his friends. We felt like repeating this prayer when we discovered the attitude of the two metropolitan medical weeklies toward the State Medical Examiners Law. We had suspected for some time past they were being weaned from the cause that they once espoused so heartily, but it was not until they both spoke out recently in such unmistakable English that we could believe that they were really opposed to the policy that many of the best medical men in the State think the wisest to pursue, at present, in regard to medical legislation. Briefly stated, this policy is about as follows: That, after several years' struggle, we have finally obtained a State Medical Examiners law; that, though it is not what was originally asked at the hands of the Legislature, it is believed to afford ample protection to the public, which is, after all, the real end sought; that it is well known that at the time it was passed we could get *no other kind of a law*, and, failing to accept this, New York could not expect to take position with the States that are improving their methods of medical education for many years; that good faith demands that the Medical Society of the State of New York shall sustain this law with its best efforts until it is fairly tested in a practical way; that then, if it shows defects they can be remedied upon application to the legislature; and that, until such time, all attempts at amendment shall be opposed with all the vigor and earnestness that a united society can present. Indeed, it may be further stated, as a general proposition, that the State Medical Society is opposed to any further medical legislation whatever at present. We have had a surfeit of this kind of business lately, and it is best to wait until we can digest and codify what we have on hand before we ask for more. We presume the Legislature will be quite willing to join issue with us on this point.

The apparent grievance of the *Medical Record* is that the City of New York was not given the majority—or indeed any—representation on the State Examining Board, as announced in the newspapers. This is reviving the old sectional question that always springs up in the minds of some whenever anything is to be done in the way of making appointments; but, in this instance, we respectfully refer the *Record* to the Board of Regents of the University of the State of New York, where is vested the appointing power.

To the *New York Medical Journal* we desire to say that we are not in the habit of speaking in these columns without authority when we refer to the actions of a body of men ; so, when we asserted in our April number that the University Bill had been introduced into the two houses of the legislature *without* the consent of the medical faculty of Niagara University, we did so advisedly. Moreover, we now re-affirm that the aforesaid faculty is not only *opposed* to that bill, but is in *favor* of the present law regarding State examination and license, desiring to have it tested before any attempt is made to amend or supplant it by further legislative action. And this, even though a single member of the faculty may have written a contrary statement to the *Journal.*

Furthermore, though the faculty in question has not yet taken *formal* action, we are informed that it will have an opportunity to do so at its next meeting. We are not aware that any " deservedly influential " members of the Erie County medical profession are to a great extent of the way of thinking on this question as is the *Journal.* We have been at some pains to inform ourselves on this subject, for we do not think it fair for the profession in this county to be misrepresented on such an important question.

